# Cost-Effectiveness of Amphotericin B Deoxycholate Versus Itraconazole for Induction Therapy of Talaromycosis in Human Immunodeficiency Virus–Infected Adults in Vietnam

**DOI:** 10.1093/ofid/ofab357

**Published:** 2021-07-05

**Authors:** James Buchanan, James Altunkaya, Nguyen Van Kinh, Nguyen Van Vinh Chau, Vo Trieu Ly, Pham Thi Thanh Thuy, Vu Hai Vinh, Doan Thi Hong Hanh, Nguyen Thuy Hang, Tran Phuong Thuy, Rogier van Doorn, Guy Thwaites, Alastair Gray, Thuy Le

**Affiliations:** 1 Health Economics Research Centre, Nuffield Department of Population Health, University of Oxford, Oxford, United Kingdom; 2 National Hospital for Tropical Diseases, Hanoi, Vietnam; 3 Hospital for Tropical Diseases, Ho Chi Minh City, Vietnam; 4 University of Medicine and Pharmacy, Ho Chi Minh City, Vietnam; 5 Bach Mai Hospital, Hanoi, Vietnam; 6 Viet Tiep Hospital, Hai Phong, Vietnam; 7 Vietnam-Sweden Uong Bi Hospital, Quang Ninh, Vietnam; 8 Oxford University Clinical Research Unit, Ho Chi Minh City, Vietnam; 9 Oxford University Clinical Research Unit, Ho Chi Minh City, Vietnam; Centre for Tropical Medicine and Global Health, Nuffield Department of Medicine, University of Oxford, United Kingdom; 10 Duke University School of Medicine, Durham, North Carolina, USA

**Keywords:** amphotericin B, cost-effectiveness, HIV, itraconazole, talaromycosis

## Abstract

**Background:**

Talaromycosis (penicilliosis) is an invasive fungal infection and a major cause of human immunodeficiency virus (HIV)–related deaths in Southeast Asia. Guidelines recommend induction therapy with amphotericin B deoxycholate; however, treatment with itraconazole has fewer toxic effects, is easier to administer, and is less expensive. Our recent randomized controlled trial in Vietnam found that amphotericin B was superior to itraconazole with respect to 6-month mortality. We undertook an economic evaluation alongside this trial to determine whether the more effective treatment is cost-effective.

**Methods:**

Resource use, direct and indirect costs, and health and quality-of-life outcomes (measured using quality-adjusted life-years [QALYs]) were evaluated for 405 trial participants from 2012 to 2016. Both a Vietnamese health service and a broader societal costing perspective were considered. Mean costs and QALYs were combined to calculate the within-trial cost-effectiveness of amphotericin vs itraconazole from both perspectives.

**Results:**

From a Vietnamese health service perspective, amphotericin increases costs but improves health outcomes compared to itraconazole, at a cost of $3013/QALY gained. The probability that amphotericin is cost-effective at a conventional (World Health Organization CHOICE) threshold of value for money is 46%. From a societal perspective, amphotericin is cost-reducing and improves outcomes compared to itraconazole, and is likely to be a cost-effective strategy at any value for money threshold greater than $0.

**Conclusions:**

Our analysis indicates that induction therapy with amphotericin is a cost-effective treatment strategy for HIV-infected adults diagnosed with talaromycosis in Vietnam. These results provide the evidence base for health care providers and policy makers to improve access to and use of amphotericin.

## Teaser

Compared to itraconazole as induction therapy for talaromycosis, amphotericin leads to more adverse events and is more expensive, but reduces mortality. Here, we show that, from a societal perspective, amphotericin is likely to be cost-effective in HIV-infected adults in Asia.

Talaromycosis (formerly penicilliosis) is an invasive fungal infection caused by the thermally dimorphic fungus *Talaromyces marneffei* that is endemic throughout Southeast Asia, southern China, and northeastern India. Patients with advanced human immunodeficiency virus (HIV) disease (CD4 count <100 cells/mm^3^) are at risk and develop disseminated infection involving the skin, lung, liver, spleen, lymphatics, bloodstream, and bone marrow [1]. Driven by the HIV pandemic, talaromycosis is now the third most common cause of HIV-related infections; it accounts for up to 25% of HIV-related hospital admissions and is a leading cause of HIV-associated bloodstream infections and death in Vietnam and southern China [2–10]. Increasingly, talaromycosis is being diagnosed in patients with immunodeficiency conditions other than HIV (including patients with interferon-γ autoantibody, autoimmune diseases, cancers, and after undergoing organ and bone marrow transplantation) [11]. Talaromycosis is also increasingly diagnosed in immigrants and in returning travelers from Southeast Asia [12]. The mortality rate on antifungal therapy is 10%–30% in HIV-infected patents [2, 3, 6–8], and 50% in non-HIV-infected patients [11].

Current treatment options in endemic regions are largely limited to 2 drugs, amphotericin B deoxycholate (hereafter, “amphotericin”) and itraconazole. Prior to the trial that underpins the economic evaluation reported in this article, international guidelines recommended induction therapy with amphotericin, delivered intravenously at a daily dose of 0.7–1 mg/kg of body weight for 2 weeks, followed by consolidation therapy with itraconazole, delivered orally at a dose of 400 mg per day for 10 weeks [13, 14]. This recommendation was informed by expert opinion and the results of a noncomparative study using this strategy in Thailand. However, in low-resource countries such as Vietnam, Myanmar, and India, itraconazole is used more frequently than amphotericin because it is widely available in oral formulation, is cheaper, and has fewer side effects.

We recently completed a randomized controlled trial comparing itraconazole vs amphotericin for induction therapy of talaromycosis in 5 hospitals in Vietnam (Itraconazole versus Amphotericin B for Penicilliosis [IVAP] trial) [15]. We found amphotericin to be superior with respect to 6-month mortality (11.3% vs 21.0% in the itraconazole group; absolute risk difference, 9.7% [95% confidence interval {CI}, 2.8%–16.6%]). Amphotericin treatment was also associated with faster clearance of fungemia, faster resolution of symptoms, and lower rates of relapse and immune reconstitution inflammatory syndrome. Although more frequent drug-related adverse events were observed in the amphotericin group, the overall incidence of serious adverse events was lower.

As amphotericin is more expensive than itraconazole, the IVAP trial findings have significant implications for limited health care budgets in HIV-endemic countries in Southeast Asia. However, there are no data on whether the more effective treatment is cost-effective. We undertook an economic evaluation alongside the IVAP trial to evaluate resource use, costs, and health outcomes associated with the 2 treatments, and combine these data in a cost-effectiveness analysis to inform treatment policy for talaromycosis.

## METHODS

Here we describe our analytical approach; how data were collected on costs, resource use, and outcomes for each treatment strategy; how missing data were addressed; and the analyses performed.

### Analytical Approach

**Figure 1.**
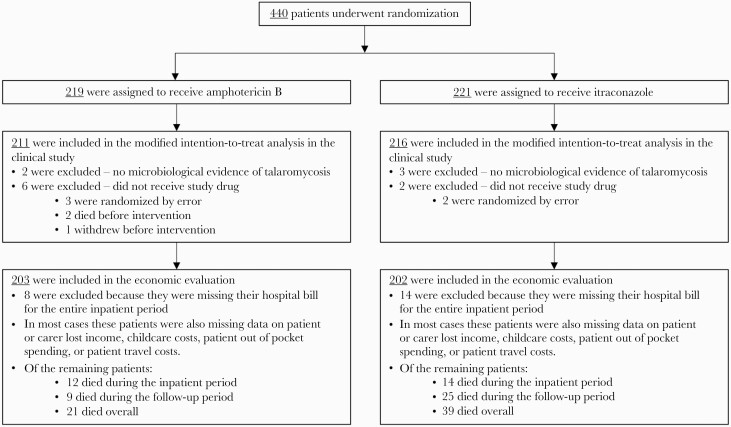
Summary of patients included in the economic evaluation.

A modified intention-to-treat approach was adopted for the economic evaluation, as per the IVAP trial. Thirteen of 440 patients initially randomized to treatment were excluded because they did not receive the study drug, or there was no microbiological evidence of talaromycosis. Twenty-two patients were excluded because their hospital bills were missing; the majority (14) were from 1 of 5 study sites. In total, 405 patients were included in this analysis (Figure 1).

The economic evaluation followed the time frame of the trial, with resource use, costs, and health and quality-of-life (QoL) outcomes evaluated for each patient from hospital admission until discharge (“inpatient period”), then until the end of 6-month follow-up (“follow-up period”). The economic evaluation results were calculated from the perspective of the Vietnamese health service and from a societal perspective, which included non–health care–related resource use. Patients were recruited from October 2012 through December 2015 with follow-up ending in June 2016.

### Resource Use and Costs

#### Health Care Resource Use and Costs.

Data on health care resource use were prospectively collected at study enrollment, end of the inpatient period, and end of follow-up. Data were collected on all laboratory and radiographic investigations, adverse events, inpatient stays, outpatient visits, treatments and interventions including blood and platelet transfusion, and emergency care. Costs were extracted from patient billing records, as no standard unit costs for Vietnamese health care were available.

Use of itraconazole and amphotericin during the inpatient period was recorded within the trial. In the itraconazole arm, patients received a loading dose of 600 mg per day for 3 days, then 400 mg per day thereafter. In the amphotericin arm, patients received 0.7 mg per kg per day. During follow-up, all patients received a consolidation dose of 400 mg itraconazole per day for 10 weeks, then 200 mg per day thereafter.

The costs associated with itraconazole and amphotericin treatment were covered by trial funding. For this analysis, we used unit costs reflecting local market prices: 18 500 Vietnamese Dong (VND) (US$0.89) per 100-mg tablet for itraconazole and 416 920 VND (US$20) per 50-mg vial of amphotericin. As the price for amphotericin was originally expressed in US dollars, we converted this into VND using the exchange rate on the date of the first hospital admission in the trial (20 846 VND to US$1) [16], to reflect local market prices at the point of drug purchase. If an entire vial was not required, the excess was discarded.

#### Non–Health Care Resource Use and Costs.

The societal analysis included the following costs (details in Supplementary Materials Part 1):

- *Patient lost income*: Data were collected on income lost during the inpatient period and days off work during the follow-up period, but no data were collected on income lost during the follow-up period. If patients provided data on income lost during the inpatient period, their daily wage rate was applied to time off work during the follow-up period; otherwise, daily wage rates by region were applied to time off work [17].
- *Carer lost income*: Data were collected on income lost by carers attending patients during the inpatient and follow-up periods.
- *Childcare costs*: Data were collected on days of childcare required during the inpatient and follow-up periods. Regional minimum wage rates in Vietnam were used as a proxy for childcare costs as in most cases the carer was a relative [18].
- *Patient out-of-pocket spending*: Data were collected on patient out-of-pocket spending during the inpatient and follow-up periods. This included spending on over-the-counter medications, user fees, food and drink (while hospitalized), and medical supplies.
- *Patient travel costs*: Data were collected on travel costs to and from hospital during the inpatient and follow-up periods.

### Health and Quality-of-Life Outcomes

All-cause mortality was recorded throughout the trial. Patients completed EQ-5D-5L health status surveys on admission (survey 1), after the 2-week inpatient period (survey 2), and at 24-week follow-up (survey 3) (Supplementary Materials Part 1). Each completed EQ-5D-5L survey yielded a health-related QoL profile, which was assigned an index value (“utility”). A recent EQ-5D-5L valuation study for Vietnam was used to calculate index values [19, 20]. Utility was set to zero from the date of death onward. Linear interpolation was used to calculate quality-adjusted survival between the dates of the 3 surveys and/or death, expressed as a fraction of 1 year in full health (quality-adjusted life-years [QALYs]). Differences in QoL between treatment arms at baseline were adjusted for observed QoL in our base-case analysis [21].

### Missing Data

The overall level of missing data was low (described in Supplementary Materials Part 2). Multiple imputation with chained equations was used to replace missing observations with predicted values based on observed data [22], using Stata version 14 software (StataCorp, College Station, Texas).

### Analyses Performed

#### Main Analysis.

**Figure 2.**
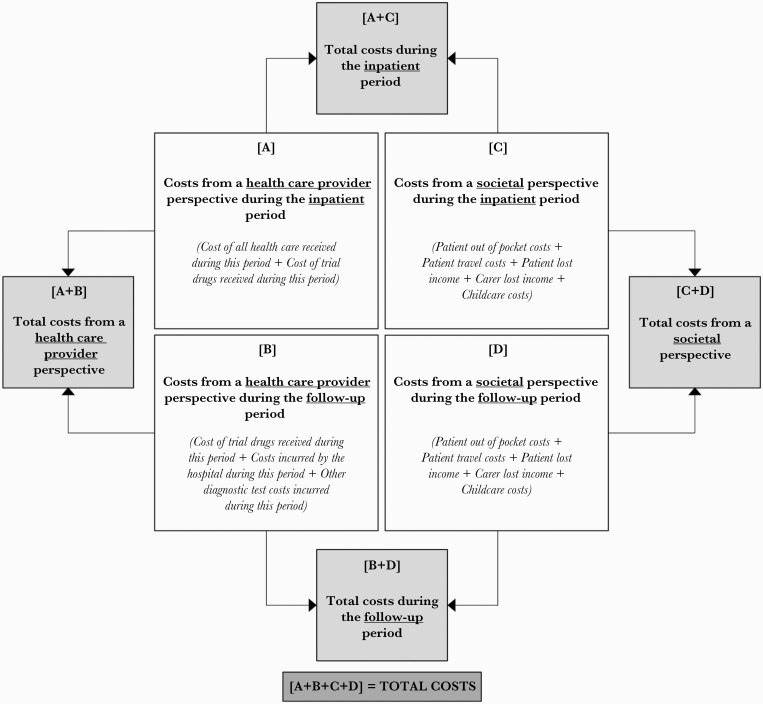
Summary of cost components by study period and costing perspective.

Cost data were summarized by costing perspective (health care provider, societal) and study period (inpatient, follow-up) (Figure 2). Within-trial costs and QALYs were not discounted due to the short time horizon of the study, as no patient had a combined inpatient and follow-up period that exceeded 1 year. Mean costs and QALYs accrued in each study arm were calculated and combined in an incremental analysis to calculate the cost-effectiveness of amphotericin vs itraconazole from both costing perspectives. Results are presented in 2016 international dollars, converted from Vietnamese Dong using the 2016 World Bank gross domestic product (GDP) purchasing power parity conversion factor for Vietnam (7315.61 VND per international dollar) [23]. We also calculated cost-effectiveness using life-years as the outcome measure (Supplementary Materials Part 7).

Uncertainty surrounding the results was evaluated using nonparametric bootstrapping. For each of the 75 imputed datasets, 1000 bootstrap resamples were drawn. The resulting cost and effect pairs were used to calculate cost-effectiveness acceptability curves that evaluated which treatment was the cost-effective strategy at a range of cost-effectiveness thresholds. The CIs for incremental cost-effectiveness ratios were calculated using the percentile approach. Assessing value for money requires a cost-effectiveness threshold, but consensus on the appropriate threshold for Vietnam is absent. We use the World Health Organization Choosing Interventions That Are Cost-Effective (WHO-CHOICE) threshold for very good value for money of annual GDP per capita in Vietnam, which in 2016 was $2171 [24, 25]. In a sensitivity analysis we also consider low and high cost-effectiveness thresholds as proposed by Woods et al [26], using an opportunity cost approach, of $144 and $982, respectively.

#### Sensitivity Analyses.

Sensitivity analyses explored the impact on our results if drug costs were 50% above or below their assumed level.

#### Lifetime Analysis.

The within-trial analysis ignores cost or outcome differences continuing beyond the trial follow-up period. To address this, we conducted a lifetime analysis that estimated costs and health outcomes accrued by patients after trial follow-up had ceased. Costs and health outcomes were then discounted to present values at a rate of 3% per annum and combined with the within-trial results to derive lifetime estimates (details are shown in Supplementary Materials Part 3).

## RESULTS

Patient characteristics are described in Supplementary Materials Part 4. Baseline differences by study arm in mean age (34 years), sex (68% male), proportion of patients who were intravenous drug users (31%), and QoL were not statistically significant.

### Resource Use and Costs

There were few significant differences in resource use by study arm across the inpatient and follow-up periods (Supplementary Materials Part 5). Healthcare–related resource use was higher for patients in the itraconazole arm during the follow-up period; however, the differences were not statistically significant.

Tables 1–3 present the cost results (see Supplementary Materials Part 6 for results in VND). From a health care provider perspective, patients in the amphotericin arm had significantly higher costs during the inpatient period (mean difference, $586 [95% CI, $239–$933]), primarily due to higher drug costs, but significantly lower costs during the follow-up period (mean difference, –$504 [95% CI, –$924 to –$84]), primarily due to fewer subsequent hospitalizations and outpatient clinic visits. Over the entire trial, patients in the amphotericin arm had slightly but not significantly higher costs from a health care provider perspective (mean difference, $82 [95% CI, –$501 to $666]) (Table 1).

**Table 1.** Mean Patient Costs From a Healthcare Provider Perspective, in 2016 International Dollars

		Study Arm: Mean (SE) ($)		
Study Timepoint	Cost Category	Amphotericin B	Itraconazole	Difference^a^, Mean (95% CI) ($)
Inpatient period (n = 405)	Diagnosis	432 (16)	428 (16)	4 (–40 to 48)
	Inpatient stays	183 (14)	171 (8)	12 (–20 to 45)
	Drugs	843 (83)	872 (88)	–30 (–267 to 208)
	Procedures and operations	12 (4)	10 (2)	2 (–6 to 10)
	Blood transfusions	227 (23)	177 (23)	51 (–13 to 114)
	Platelets	53 (19)	79 (28)	–26 (–92 to 40)
	Other costs	105 (7)	90 (7)	15 (–4 to 35)
	Trial drugs	737 (16)	180 (9)	557 (520–594)^b^
	Total—inpatient period	2592 (126)	2006 (124)	586 (239–933)^b^
Follow-up period (n = 379)	Trial drugs	1048 (22)	1006 (24)	42 (–23 to 106)
	Hospital costs	361 (82)	895 (195)	–534 (–952 to –117)^c^
	Other test costs	1 (0)	3 (1)	–2 (–4 to –1)^b^
	Other diagnostic costs	50 (2)	59 (3)	–9 (–16 to –1)^c^
	Total—follow-up period	1459 (85)	1963 (195)	–504 (–924 to –84)^c^
	Total—inpatient and follow-up period	4052 (164)	3969 (247)	82 (–501 to 666)

Abbreviations: CI, confidence interval; SE, standard error.

^a^Difference = amphotericin minus itraconazole.

^b^Significant at 1% level.

^c^Significant at 5% level.

**Table 2.** Mean Non–Health Care Costs, in 2016 International Dollars

		Study Arm: Mean (SE) ($)		
Study Timepoint	Cost Category	Amphotericin B	Itraconazole	Difference^a^, Mean (95% CI) ($)
Inpatient period (n = 405)	Patient out-of-pocket costs	450 (36)	477 (42)	–27 (–135 to 80)
	Patient travel costs	97 (11)	114 (15)	–17 (–55 to 20)
	Patient lost income	273 (37)	299 (50)	–26 (–148 to 96)
	Carer lost income	196 (22)	202 (20)	–6 (–65 to 52)
	Childcare costs	79 (9)	74 (10)	5 (–21 to 31)
	Total—inpatient period	1095 (69)	1166 (87)	–71 (–289 to 146)
Follow-up period (n = 379)	Patient out-of-pocket costs	228 (50)	210 (37)	19 (–104 to 138)
	Patient travel costs	193 (24)	187 (21)	6 (–57 to 69)
	Patient lost income	134 (24)	198 (56)	–64 (–183 to 55)
	Carer lost income	82 (24)	102 (25)	–20 (–89 to 49)
	Childcare costs	45 (10)	70 (20)	–25 (–68 to 18)
	Total—follow-up period	682 (73)	765 (94)	–84 (–316 to 148)
	Total—inpatient and follow-up period	1777 (107)	1932 (139)	–155 (–498 to 188)

Abbreviations: CI, confidence interval; SE, standard error.

^a^Difference = amphotericin minus itraconazole.

**Table 3.** Mean Societal (Healthcare Provider and Non–Health Care) Costs per Patient, in 2016 International Dollars

	Study Arm: Mean (SE) ($)		
Cost Category	Amphotericin B	Itraconazole	Difference^a^, Mean (95% CI) ($)
Inpatient period	3688 (156)	3173 (178)	515 (49–980)^b^
Follow-up period	2141 (129)	2729 (230)	–587 (–1104 to –71)^b^
Total—inpatient and follow-up period	5829 (225)	5901 (316)	–73 (–831 to 686)

Abbreviations: CI, confidence interval; SE, standard error.

^a^Difference = amphotericin minus itraconazole.

^b^Significant at 5% level.

Non–health care costs were slightly but not significantly lower among patients in the amphotericin arm, during both the inpatient and follow-up periods, with a nonsignificant mean difference over the entire trial of –$155 (95% CI, –$498 to $188) (Table 2).

Combining health care and non–health care costs in a societal perspective (Table 3), patients in the amphotericin arm had significantly higher costs during the inpatient period (mean difference, $515 [95% CI, $49–$980]), but significantly lower costs during the follow-up period (mean difference, –$587 [95% CI, –$1104 to –$71]). Over the entire trial the mean difference was –$73 (95% CI, –$831 to $686).

#### Health and QoL Outcomes.

**Figure 3.**
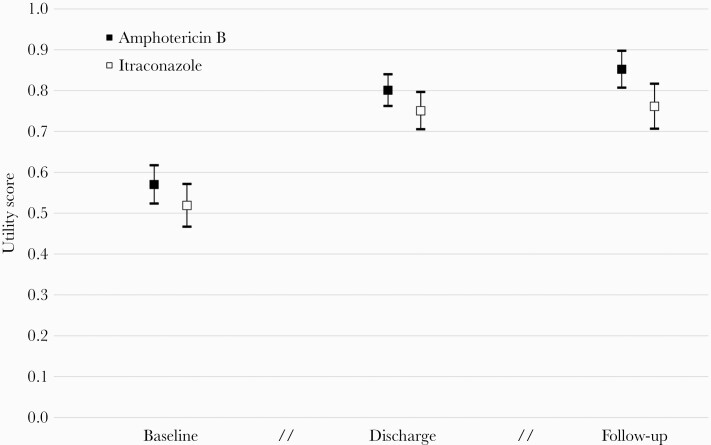
Mean utility scores and 95% confidence intervals by study time point and study arm. Breaks indicate that intervals between study time points are not equal.

Patients in the amphotericin arm generally reported better QoL at both discharge and end of follow-up (Supplementary Table 7.1). Mean utility scores were significantly higher for amphotericin than for itraconazole at all 3 study timepoints (Figure 3). Adjusted for small differences in baseline QoL, patients in the amphotericin arm had significantly higher quality-adjusted survival (mean QALY difference, 0.027 [95% CI, .002–.052]; Supplementary Table 7.2). Patients in the amphotericin arm also gained significantly more life-years (mean difference, 0.016 life-years [95% CI, .001–.031]; Supplementary Materials Part 7).

#### Cost-Effectiveness Analysis.

From a health care provider perspective, amphotericin improves health outcomes compared to itraconazole at a cost of $3013 per QALY gained (Table 4). When judged against the WHO-CHOICE cost-effectiveness threshold of $2171, and when taking into account uncertainty surrounding these results (Supplementary Figures 8.1 and 8.2), there is a 46% probability that amphotericin is the cost-effective strategy. When a societal perspective is considered, amphotericin reduces costs and improves health outcomes, with a 63% probability of being cost-effective. When life-years were used as the outcome measure, the cost per life-year gained for amphotericin vs itraconazole was $5079 from a health care provider perspective (Supplementary Materials Part 7).

**Table 4.** Cost-Effectiveness Analysis Results for Amphotericin Versus Itraconazole, in 2016 International Dollars

Costing Perspective	Incremental Costs, $	Incremental QALYs	Cost per QALY Gained, $	95% CI for Cost- Effectiveness Ratio, $	Probability of Amphotericin Being Cost-Effective at WHO-CHOICE Per-Capita Income Threshold ($2171), %
Adjusted for difference in baseline quality of life between arms					
Healthcare provider costs only	82	0.027	3013	Cost saving to 51 913 per QALY gained	46
Societal costs	–73	0.027	NA^a^	Cost saving to 51 360 per QALY gained	63
No adjustment for difference in baseline quality of life between arms					
Healthcare provider costs only	82	0.034	2438	Cost saving to 41 999 per QALY gained	48
Societal costs	–73	0.034	NA^a^	Cost saving to 38 110 per QALY gained	65

Incremental figures = amphotericin minus itraconazole.

Abbreviations: CI, confidence interval; NA, not applicable; QALY, quality-adjusted life-year; WHO-CHOICE, World Health Organization Choosing Interventions That Are Cost-Effective.

^a^Amphotericin reduces costs and improves health outcomes.

#### Sensitivity and Lifetime Analyses.

When the amphotericin cost is reduced by 50%, the probability that amphotericin is the cost-effective treatment strategy increases from 46% to 87% (health care provider perspective) and from 63% to 90% (societal perspective). Changes in the cost of itraconazole have a smaller impact on the cost-effectiveness analysis results (Supplementary Materials Part 9).

The probability that amphotericin is cost-effective from a health system perspective using the Woods et al cost-effectiveness threshold is 39% at the lower threshold and 42% at the higher threshold (Supplementary Materials Parts 8 and 9). From a societal perspective, the probability that amphotericin is the cost-effective treatment strategy is 58% at the lower and 60% at the higher Woods threshold.

When the trial results are extended to a lifetime perspective (Supplementary Materials Part 3), amphotericin increases costs (mean difference, $725 [95% CI, $458–$996]) and improves health outcomes (mean QALY difference, 1.06). The discounted lifetime cost-effectiveness from a societal perspective is $655/QALY gained, below the WHO cost-effectiveness threshold ($2171).

## DISCUSSION

In this randomized trial-based economic evaluation of the use of amphotericin vs itraconazole for induction therapy of HIV-associated talaromycosis in Vietnam, we found that, from a health care perspective, amphotericin increases costs and improves health outcomes compared to itraconazole. The probability that amphotericin is the cost-effective treatment strategy at the WHO-CHOICE cost-effectiveness threshold is 46%. However, viewed from a societal perspective that includes broader costs to patients and society, amphotericin is cost-saving and improves outcomes compared to itraconazole, and is therefore likely to be a cost-effective strategy at any threshold greater than $0. The higher costs of amphotericin during the inpatient period are offset by lower health care costs and reduced lost income during the follow-up period, driven by lower incidence of disease complications.

Our results complement the main findings of the clinical trial, which found that amphotericin therapy was associated with reduced mortality, faster resolution of symptoms, and lower rates of disease complications. This analysis provides reliable evidence that induction therapy with amphotericin is likely to be a cost-effective treatment strategy for HIV-infected adults diagnosed with talaromycosis in countries with similar health care and economic settings in Southeast Asia. This conclusion is greatly strengthened when taking into account broader societal cost savings, the longer-term benefits of this therapy, and trends in reductions in the cost of amphotericin and duration of treatment since our study was conducted. In particular, the statistically significant survival benefit reported in the main trial results translates in our extrapolation into a substantial difference in life expectancy and hence a much lower lifetime cost-effectiveness ratio.

The cost-effectiveness of amphotericin induction therapy can be compared with that of other interventions considered cost-effective in Vietnam and Southeast Asia to reduce HIV-related morbidity and mortality, while bearing in mind differences in study methods, dates, perspectives, and settings: for example, HIV prevention programs in Vietnam ($2344 per death averted) [27]; methadone maintenance treatment to prevent HIV acquisition among injection drug users in Vietnam ($1964 per QALY gained) [28]; screening HIV-infected adults in Vietnam for cryptococcal infection using an antigen test ($119–$190 per life-year gained) [29]; strategies for cryptococcosis prevention in HIV-infected patients in Cambodia ($180–$1538 per life-year gained) [30]; and isoniazid primary prophylaxis for tuberculosis in HIV-infected individuals in southern India ($1490–$3120 per year of life saved) [31].

Although we show that amphotericin is likely to be a cost-effective treatment strategy, it is currently not widely available in Southeast Asia, has higher upfront drug costs, and requires higher levels of knowledge and skills in drug administration, and monitoring and management of side effects. Widespread adoption would require the health sector to preallocate financial resources to drug procurement and training of health care staff in drug administration and monitoring, which requires careful budget impact analyses. Implementation would be challenging, particularly in district- and commune-level hospitals where the volume of talaromycosis patients is lower and nursing skills and facilities for intravenous infusion and monitoring are limited. Whether current guidelines on induction therapy in talaromycosis are actually followed will also affect the affordability of changing health care practice.

Our study has several strengths. We used detailed data on costs and morbidity and mortality outcomes carefully collected within a randomized controlled trial, minimizing unobserved bias and reducing the number of assumptions required. Data on costs incurred by patients and carers outside the health sector were included, permitting a societal analytical perspective. QoL data were collected directly from patients, not derived from previous studies, and we were able to use locally estimated utility values. We also fully considered patient- and parameter-level uncertainty, and undertook an illustrative extrapolation of the cost-effectiveness estimates beyond the timeframe of the clinical study. Finally, IVAP was a pragmatic trial conducted in 5 central and provincial hospitals; hence, the clinical and cost-effectiveness results are potentially generalizable across Vietnam and similar health care settings in the wider region. While no standard national unit costs for Vietnamese health care were available, comprehensive billing records were available to generate accurate costs for our patient sample. Our results are less generalizable to higher-resourced countries such as Singapore or Thailand, where access to antiretroviral therapy and health care financing systems and costs are substantially different.

Our study also has some limitations. First, because we quantified health outcomes using QALYs, comparisons with studies that use disability-adjusted life-years (DALYs)—a common metric in economic evaluations in developing countries—are more difficult [32]. However, a recent study concluded that the choice of approach to compute health benefits seldom alters the conclusions of such studies. The authors further noted that any uncertainty arising from using either QALYs or DALYs is unlikely to be greater than that due to other study variables, such as the variations in drug cost considered in our analysis [33]. A further recent study supports this conclusion [34]. Second, our lifetime analysis was relatively simple and excludes some future lifetime medical costs in later years of life. However, the main variable driving these long-term estimates was differences in the numbers of patients alive in each trial arm at end of follow-up, a strong position from which to assume some continuing benefit. Third, we acknowledge that costs generated from billing records may not fully generalize to reflect national health care opportunity costs. However, in the absence of standard unit costs for Vietnamese health care resources, our costing approach gives the best available approximation. We further acknowledge that the economic perspective may vary between rural and urban patients; however, we did not collect reliable data to compare these perspectives. Fourth, although the overall level of missing data was low, we acknowledge that alternative analytical choices to implement the multiple imputation of missing data within a bootstrapped analysis may affect the magnitude of decision uncertainty [35]. Finally, although the trial protocol stipulated that all patients must remain in hospital for the 14-day duration of induction therapy, some patients in the itraconazole arm might in practice have been judged well enough for early discharge on oral itraconazole therapy. Inpatient costs accrued by patients in the itraconazole arm could therefore be overestimated.

In conclusion, our study provides new trial-based evidence that induction therapy using amphotericin is likely to be a cost-effective treatment strategy for HIV-infected adults diagnosed with talaromycosis in Vietnam and similar settings in Southeast Asia. Although the alternative treatment—itraconazole—is cheaper, amphotericin leads to lower health care costs and reduced income losses, and is associated with improved quality and quantity of life. Our findings support current treatment guidelines and augment the evidence base for the health care sector to invest in improving access to current and new formulations of amphotericin for HIV patients diagnosed with talaromycosis across Southeast Asia.

## Supplementary Data

Supplementary materials are available at *Open Forum Infectious Diseases* online. Consisting of data provided by the authors to benefit the reader, the posted materials are not copyedited and are the sole responsibility of the authors, so questions or comments should be addressed to the corresponding author.

ofab357_suppl_Supplementary_Materials

## Notes

***Author contributions.*** A. G. and T. L. conceived, designed, and received the funding for the study. N. V. K., N. V. V. C., V. T. L., P. T. T. T., V. H. V., D. T. H. H., N. T. H., and T. P. T. collected the data. N. T. H. and T. P. T. cleaned the data. J. B. and J. A. carried out the analyses. J. B., J. A., G. T., A. G., and T. L. interpreted the results. J. B. prepared the first draft of the manuscript. R. v. D., G. T., A. G., and T. L. provided high-level input to the first draft. All authors critically revised the manuscript and approved the final version.

***Acknowledgments.*** We thank the patients and their family members for their participation and dedication to the IVAP trial. We thank the staff at the Hospital for Tropical Diseases in Ho Chi Minh City, the National Hospital for Tropical Diseases in Hanoi, Bach Mai Hospital in Hanoi, Viet Tiep Hospital in Hai Phong, and Uong Bi Hospital in Quang Ninh for their support and participation in the IVAP trial.

***Patient consent statement.*** Written informed consent was obtained from all the patients or their representatives. The protocol was approved by the independent ethics committee at each participating hospital, by the Vietnam Ministry of Health, and by the Oxford University Tropical Research Ethics Committee. The Vietnam Ministry of Health and an independent data monitoring and ethics committee oversaw the safety of the trial.

***Disclaimer.*** The funders had no role in study design, data collection and analysis, decision to publish, or preparation of the manuscript.

***Financial support*.** This work was supported by funding from the Medical Research Council, the Department of International Development, and the Wellcome Trust, all in the UK (G1100682). The funders had no role in study design, data collection and analysis, decision to publish, or preparation of the manuscript.

***Potential conflicts of interest.*** All authors: No reported conflicts of interest.
