# Supplementary material for: Cost-Effectiveness of Amphotericin B Deoxycholate Versus Itraconazole for Induction Therapy of Talaromycosis in Human Immunodeficiency Virus–Infected Adults in Vietnam
Source: Open Forum Infect Dis. 2021 Jul 5;8(7):ofab357. doi: 10.1093/ofid/ofab357 (PMC8320272; doi:10.1093/ofid/ofab357)
Supplement: ofab357_suppl_Supplementary_Materials [file ofab357_suppl_supplementary_materials.docx]

**Cost-effectiveness of amphotericin B deoxycholate versus itraconazole for induction therapy of talaromycosis in HIV-infected adults in Vietnam**

**Running title:** Talaromycosis therapy cost-effectiveness

**Supplementary materials**

**Author list**

1. James Buchanan, Health Economics Research Centre, Nuffield Department of Population Health, University of Oxford, Oxford, United Kingdom.
2. James Altunkaya, Health Economics Research Centre, Nuffield Department of Population Health, University of Oxford, Oxford, United Kingdom
3. Nguyen Van Kinh, National Hospital for Tropical Diseases, Hanoi, Vietnam.
4. Nguyen Van Vinh Chau, Hospital for Tropical Diseases, Ho Chi Minh City, Vietnam.
5. Vo Trieu Ly, University of Medicine and Pharmacy, Ho Chi Minh City, Vietnam.
6. Pham Thi Thanh Thuy, Bach Mai Hospital, Hanoi, Vietnam.
7. Vu Hai Vinh, Viet Tiep Hospital, Hai Phong, Vietnam.
8. Doan Thi Hong Hanh, Vietnam-Sweden Uong Bi Hospital, Quang Ninh, Vietnam.
9. Nguyen Thuy Hang, Oxford University Clinical Research Unit, Ho Chi Minh City, Vietnam.
10. Tran Phuong Thuy, Oxford University Clinical Research Unit, Ho Chi Minh City, Vietnam.
11. Rogier van Doorn, Oxford University Clinical Research Unit, Ho Chi Minh City, Vietnam; Centre for Tropical Medicine and Global Health, Nuffield Department of Medicine, University of Oxford, United Kingdom
12. Guy Thwaites, Oxford University Clinical Research Unit, Ho Chi Minh City, Vietnam; Centre for Tropical Medicine and Global Health, Nuffield Department of Medicine, University of Oxford, United Kingdom
13. Alastair Gray*, Health Economics Research Centre, Nuffield Department of Population Health, University of Oxford, Oxford, United Kingdom.
14. Thuy Le*, Duke University School of Medicine, Durham, NC, USA

**Corresponding author**

James Buchanan, Health Economics Research Centre, Nuffield Department of Population Health, University of Oxford, Oxford, United Kingdom. [james.buchanan@dph.ox.ac.uk](mailto:james.buchanan@dph.ox.ac.uk). +44 1865 289262

**Alternate corresponding author**

Thuy Le, Duke University School of Medicine, Durham, NC, USA. [thuy.le@duke.edu](mailto:thuy.le@duke.edu). +1 919.668.5053

**Contents**

[Part 1: Further data on costs, health and quality of life outcomes 3](#_Toc74263255)

[Part 2: Missing data 5](#_Toc74263256)

[Part 3: Incorporating costs and health outcomes beyond the trial follow-up period 7](#_Toc74263257)

[Part 4: Patient characteristics 12](#_Toc74263258)

[Part 5: Resource use by patients during the inpatient and follow-up periods of the trial 13](#_Toc74263259)

[Part 6: Costs in Vietnamese Dong 17](#_Toc74263260)

[Part 7: Quality of life and health outcomes results 19](#_Toc74263261)

[Part 8: Cost-effectiveness acceptability curves 21](#_Toc74263262)

[Part 9: Sensitivity analysis results 23](#_Toc74263263)

[References 25](#_Toc74263264)

# Part 1: Further data on costs, health and quality of life outcomes

Costs

*Patient lost income*

For some patients, regional wage rates were applied to time taken off work during the follow-up period. These rates were based on data on monthly average income per capita from the General Statistics Office of Vietnam.[1] Data were only available for 2012 and 2014, so 2012 data were applied in 2013 and 2014 data were applied in 2015. The following monthly figures were available:

| **Region** | **Year (figures are in Vietnamese Dong)** | |
| --- | --- | --- |
|  | **2012** | **2014** |
| Hanoi | 2945000 | 4113000 |
| Quang Ninh | 2557000 | 3053000 |
| Hai Phong | 2526000 | 3923000 |
| Ho Chi Minh City | 3653000 | 4840000 |

These were converted into daily rates using information from the IVAP dataset on the average number of days worked per week.

*Childcare costs*

Regional minimum wage rates in Vietnam were used as a proxy for childcare costs as in most cases the carer was a relative. Data were available on these wage rates for three years: 2012, 2013 and 2014 onwards.[2-4] These rates were specified for four regions, of which regions 1 (Hospital for Tropical Diseases, National Hospital for Tropical Diseases, Bach Mai Hospital, Viet Tiep General Hospital) and 3 (Vietnam-Sweden Uong Bi Hospital) were relevant for this analysis.

| **Region** | **Year (figures are in Vietnamese Dong)** | | | |
| --- | --- | --- | --- | --- |
|  | **2012** | **2013** | **2014** | **2015** |
| 1 | 20000000 | 2350000 | 2700000 | 3100000 |
| 2 | 1550000 | 1800000 | 2100000 | 2400000 |

These were converted into daily rates using information from the IVAP dataset on the average number of days worked per week.

Health and quality of life outcomes

Data on mortality during the inpatient and follow-up periods were collected within the trial. Patients also completed EQ-5D-5L health status surveys on admission (Survey 1), at the end of the two-week inpatient period (Survey 2), and at the end of the 24-week (182 day) follow-up period (Survey 3). Some variation was observed in terms of completion date. For example, for Survey 1, 53% of surveys were completed in the 2 days following admission. Similarly, for Survey 2, 47% of surveys were completed between 12-16 days after admission and for Survey 3, 33% of surveys were completed between 175-189 days after admission. Three alternative approaches were considered to accommodate these variations in completion date. In the main analysis, we assumed that survey 1 was completed on the admission date, survey 2 was completed on the discharge date, and survey 3 was completed at the end of the follow-up period. Two alternative approaches were then considered. In approach 1, we assumed that the three surveys were completed at the same time points by each patient (Day 0, then 14 days after admission, then 182 days after admission). In approach 2, we calculated quality of life using the recorded dates for each survey. **Table S7.2** reports the results of these three approaches.

# Part 2: Missing data

The overall level of missing data in the IVAP dataset was low. Data on the total cost of healthcare received during the inpatient period were missing for 3.6% of patients. Data to calculate utility scores were missing or incomplete for 5.5% of patients on admission, 4.6% at end of inpatient period, and 8.1% at end of follow-up. The adjustments made to deal with missing data are described below.

*Missing EQ-5D-5L survey dates*

EQ-5D-5L utility scores were imputed when these data were missing (see the main paper for further details of the approach taken for multiple imputation). To use these scores to calculate quality-adjusted life-years, dates for the missing EQ-5D surveys had to be specified. The following assumptions were made regarding these dates:

- Admission dates were reported for all of the patients (n=17) who did not complete Survey 1. It was therefore assumed that this date was also the date of their first survey.
- Discharge dates were reported for all of the patients (n=16) who did not complete Survey 2. It was therefore assumed that this date was also the date of their second survey.
- Admission dates were reported for all of the patients (n=26) who did not complete Survey 3. It was therefore assumed that the date of their third survey was 182 days after the date of Survey 1.

*Missing EQ-5D utility scores due to study withdrawal or death*

Responses were not recorded for Surveys 2 and 3 for some patients due to study withdrawal or death. This missing data was managed as follows:

- For patients who died during the inpatient period, the date of Survey 2 was set as their date of death, with an assumed utility score of zero. The date of Survey 3 was set as 182 days after the date of Survey 1, again with an assumed utility score of zero.
- For patients who died during the follow-up period, the date of Survey 3 was set as their date of death, with an assumed utility score of zero.
- EQ-5D survey responses were not recorded for two patients due to study withdrawal:
  - Patient 20-007:
    - Admission date: 25/12/12
    - Survey 1: 26/12/12
    - Study withdrawal: 04/01/13
    - Death: 01/03/13
    - Action: The date of Survey 2 was assumed to be the date of death. The date of Survey 3 was set as 182 days after the date of Survey 1. A utility score of zero was assumed in both cases.
  - Patient 20-008:
    - Admission date: 12/01/13
    - Survey 1: 14/01/13
    - Survey 2: 24/01/13
    - Study withdrawal: 24/01/13
    - Date of last visit: 30/06/13
    - Action: Their last visit took place towards the end of the follow-up period. It was assumed that this was the date of Survey 3. The utility score associated with this survey was then imputed.

*Mean imputation*

In most cases, multiple imputation was used to deal with missing EQ-5D utility scores (see the next section). However, two patients were missing just one EQ-5D-5L domain score. To avoid increasing the complexity and running time of the multiple imputation analysis, this missing data was managed using mean imputation, as follows:

- Patient 20-003: The anxiety/depression domain level was missing for Survey 1. Mean imputation conditional on the scores reported for the other four domains for this patient returned an average score of 3. This value was therefore assumed for this domain for this patient.
- Patients 20-003 and 20-080: The anxiety/depression domain level was missing for Survey 2. Following the same approach as above, a value of 2 was assumed for this domain for patient 20-003 and a value of 1 was assumed for patient 20-080.

*Multiple imputation*

Following the above adjustments, we used multiple imputation to replace the remaining missing observations with values that were predicted based on the observed data.[5] An exploratory analysis initially considered the potential drivers of missingness in these variables, then the association between missingness and potential explanatory variables was evaluated using logistic regression, with significance tested at the 5% level.[5] Differences in missingness by study arm were explored, and four variables were identified as drivers of missingness: study site, patient age, patient sex and intravenous drug user status. The multiple imputation was conducted in Stata 14 (StataCorp. 2015. *Stata Statistical Software: Release 14*. College Station, TX: StataCorp LP) using chained equations. Prediction equations were defined for imputed variables using logit regression or prediction mean matching (PMM). In the case of PMM, the number of closest observations (nearest neighbours) from which to draw imputed values was specified individually for each imputed variable, following full consideration of issues surrounding variability and bias. The number of imputations performed was set equal to the percentage of cases with some missing data (75%). All imputations were performed separately for each study arm.

# Part 3: Incorporating costs and health outcomes beyond the trial follow-up period

The within-trial analysis will underestimate any differences in lifetime quality-adjusted survival across the two treatment groups. Given this, we also extended our analysis to incorporate the costs and health outcomes that could be accrued by patients beyond the trial follow-up period, then considered the consequences of this extension for the economic evaluation results. In brief, we calculated the HIV-adjusted life expectancy of all patients in the within-trial analysis, divided this into time spent in different health states (e.g. asymptomatic, CD4 count <200 cells/mm^3^) using trial data, adjusted for quality of life, then calculated the healthcare and non-healthcare related costs accrued in these health states. Detailed methods are described below, followed by the results of this approach.

Methods

The following steps were taken to undertake this analytical extension.

1. A dataset was created containing the 405 patients who featured in the main health economic analysis (203 patients in the amphotericin arm, 202 patients in the itraconazole arm). This dataset included the patients who died during the main trial period (21 in the amphotericin arm, 39 in the itraconazole arm). These patients accrued no quality-adjusted life-years (QALYs) or costs beyond the end of the trial period, but were included in this analysis to ensure that the mean cost and QALY calculations were accurate. Of note, the average age of patients who survived beyond the end of the trial period (and thus accrued additional costs and QALYs in this analysis) was significantly lower than the average age of those patients who died during the trial period (33.67 years versus 36.58 years, mean difference 2.91 years, 95% confidence interval 0.92-4.91 years).
2. Data on patient age was combined with Vietnamese life table data to calculate the number of life years remaining for each patient **(A)**.[6]
3. **(A)** was weighted to account for lower life expectancy in patients with HIV, then converted into life days remaining by multiplying this figure by 365.25 **(B)**. The age- and sex-specific weights that were applied were based on data on the life expectancy of patients receiving combination antiretroviral therapy with CD4 counts less than 50 cells/mm^3^ in Uganda.[7]
4. It was assumed that during the time period defined in **(B)**, patients could either be:
   1. Asymptomatic;
   2. Have a CD4 count below 200;
   3. Have AIDS, or
   4. Be dead.
5. It was further assumed that all patients would exit the follow-up period with a CD4 count of less than 200 cells/mm^3^.
6. Data from the IVAP study was used to calculate the mean number of days that it would take for CD4 counts to rise above this level, by study arm **(C)**. For patients receiving amphotericin, this was 647 days; for patients receiving itraconazole this was 614 days.
7. Data from the IVAP study was also used to calculate the mean number of days that patients would have AIDS before dying, by study arm **(D)**. For patients receiving amphotericin, this was 616 days; for patients receiving itraconazole this was 430 days.
8. The number of asymptomatic days experienced by each patient **(E)** was calculated as **(B)** – **(C)** – **(D)**.
9. The data on life expectancy calculated in **(C)**, **(D)** and **(E)** was then adjusted to account for quality of life during these time periods. This adjustment was undertaken using EQ-5D-5L data from a survey of 1,016 patients with HIV/AIDS in Vietnam.[8] EQ-5D-5L utility scores of 0.73 (asymptomatic patients), 0.58 (patients with CD4 counts less than 200 cells/mm^3^), and 0.62 (patients with AIDS) were applied.
10. It was assumed that all patients would be maintained on itraconazole (200mg/day) until their CD4 count rises above 200 cells/mm^3^, based on Vietnamese guidelines.[9] It was further assumed that once a patient’s CD4 count rises above 200 cells/mm^3^, patients are asymptomatic and receive no further itraconazole treatment.
11. The cost of itraconazole treatment was calculated using a unit cost of 18,500 VND per 100mg tablet, as per the main analysis.
12. Patients with AIDS accrued a daily cost of 10,552 VND. This cost was informed by a study by Tran *et al.* that estimated the costs incurred by patients with AIDS in Vietnam.[10] This figure includes the costs associated with HIV/AIDS-related services such as viral load tests, hospital admission fees, the diagnosis and treatment of comorbid diseases and non-medical expenditures such as transport and accommodation. This figure was estimated in 2011 but was not adjusted for this analysis as a healthcare inflation index could not be identified for Vietnam.
13. Mean costs and QALYs per patient were then calculated, by study arm, and used to consider the cost-effectiveness of amphotericin and itraconazole from a lifetime costing perspective.
14. Both health and cost outcomes were each discounted at a rate of 3% per annum.

Results

*QALY calculations*

**Table S3.1** summarises the calculations that were performed to estimate the mean QALYs gained in each study arm in the lifetime analysis, then combines these estimates with those for the within-trial analysis to generate an overall estimate of quality-adjusted life expectancy across both analytical timeframes.

Mean life years remaining (both before and after adjustment for HIV status) were higher in the amphotericin arm than in the itraconazole arm. This difference was entirely due to the higher within-trial mortality in the itraconazole arm. It took significantly longer for the CD4 counts of patients in the amphotericin arm to rise above 200 cells/mm^3^, and these patients also spent more time in the asymptomatic state and significantly more time living with AIDS. When this time was adjusted for quality of life, patients in the amphotericin arm accrued significantly more QALYs (12.29 versus 11.26, 95% confidence interval for difference 0.03 to 2.03). Across both the within-trial and lifetime analysis, patients in the amphotericin arm accrued more QALYs. With no discounting of future health outcomes, the amphotericin arm accrued 12.67 QALYs compared to 11.61 in the Itraconazole arm (mean difference 1.06). When discounting future health outcomes at 3% per annum, the difference in QALYs shrinks between arms, with amphotericin accruing 8.97 QALYs compared to 8.19 QALYS for Itraconazole (mean difference 0.77).

**Table S3.1: QALY calculations for the lifetime analysis**

| **Measure** | **Study arm: Mean (SE)** | | **Mean difference**  **(95% confidence interval)** |
| --- | --- | --- | --- |
|  | **Amphotericin** | **Itraconazole** |  |
| A: Life years remaining (no adjustment for lower life expectancy in patients with HIV) | 40.67  (1.10) | 37.40  (1.39) | 3.27  (-0.21 to 6.75) |
| B: Life years remaining (adjusted for lower life expectancy in patients with HIV) | 17.39  (0.44) | 15.85  (0.56) | 1.54  (0.13 to 2.95) |
| C: Life days remaining (B * 365.25) | 6,353.18 (160.94) | 5,790.36 (206.01) | 562.82  (49.20 to 1,076.44) |
| D: Number of days until CD4 counts rose above 200 cells/mm^3^ | 580.49  (13.87) | 495.62  (17.10) | 84.87  (41.61 to 128.14) |
| E: Number of days in an asymptomatic state | 5,220.07  (135.69) | 4,947.42  (177.95 | 272.66  (-166.98 to 712.29) |
| F: Number of days living with AIDS | 552.62  (13.21) | 347.33  (11.98) | 205.29  (170.22 to 240.36) |
| G: Number of days until CD4 counts rose above 200 cells/mm^3^ – quality-adjusted | 336.68  (8.05) | 287.46  (9.92) | 49.23  (24.13 to 74.32) |
| H: Number of days in an asymptomatic state – quality-adjusted | 3,810.65  (99.06 | 3,611.61  (129.90) | 199.04  (-121.90 to 519.97) |
| I: Number of days living with AIDS – quality-adjusted | 342.62  (8.19) | 215.34  (7.43) | 127.28  (105.54 to 149.02) |
| J: Overall quality-adjusted life expectancy in post-trial extrapolation in days (G+H+I) | 4,489.96  (114.16) | 4,114.41  (146.62) | 375.55  (10.46 to 740.63) |
| K: Overall quality-adjusted life expectancy in post-trial extrapolation in years | 12.29  (0.31) | 11.26  (0.40) | 1.03  (0.03 to 2.03) |
| L: Quality-adjusted life expectancy in within-trial analysis in years | 0.38  (0.01) | 0.35  (0.01) | 0.03  (0.01 to 0.06) |
| M: Undiscounted quality-adjusted life expectancy: within-trial analysis and post-trial extrapolation (K+L)^a^ | 12.67 | 11.61 | 1.06 |
| *N: Discounted quality-adjusted life expectancy: within-trial analysis and post-trial extrapolation* ^a b^ | *8.97* | *8.19* | *0.77* |

SE = standard error. Difference = Amphotericin minus Itraconazole. ^a^ Standard errors and confidence intervals cannot be calculated for combined quality-adjusted life years as QALYs for the within-trial analysis were calculated using multiple imputation, whereas those for the lifetime analysis were not. ^b^ Discounted at 3% per annum

*Cost calculations*

**Table S3.2** summarises the mean costs accrued by patients in the two study arms in the within-trial analysis and the lifetime analysis.

**Table S3.2: Costs accrued per patient in the within-trial analysis and lifetime analysis, in 2016 International dollars**

| **Analysis** | **Study arm: Mean (SE) costs per patient ($)** | | **Mean difference**  **(95% confidence interval)** |
| --- | --- | --- | --- |
|  | **Amphotericin** | **Itraconazole** |  |
| Within-trial analysis: healthcare provider costs (A) | 4,052  (164) | 3,969  (247) | 82  (-501 to 666) |
| Within-trial analysis: societal (healthcare provider and non-health care) costs (B) | 5,829  (225) | 5,901  (316) | -73  (-831 to 686) |
| Post-trial extrapolation costs (C) | 3,733  (89) | 3,008  (104) | 725  (458 to 996) |
| Lifetime analytical extension 1: Undiscounted healthcare provider costs and post-trial extrapolation costs (A+C) ^a^ | 7,785 | 6,977 | 808 |
| *Lifetime analytical extension 1: Discounted ^a b^* | *7,158* | *6,504* | *654* |
| Lifetime analytical extension 2: Societal (healthcare provider and non-health care) costs and post-trial extrapolation costs (B+C) ^a^ | 9,562 | 8,909 | 653 |
| *Lifetime analytical extension 2: Discounted ^a b^* | *8,882* | *8,378* | *504* |

SE = standard error. Difference = Amphotericin minus Itraconazole. ^a^ Standard errors and confidence intervals cannot be calculated for combined costs as costs for the within-trial analysis were calculated using multiple imputation, whereas those for the lifetime analysis were not. ^b^ Discounted at 3% per annum

Patients in the amphotericin arm accrued more costs in the lifetime analysis for two reasons. First, it took longer for their CD4 counts to rise above 200 cells/mm^3^, hence they accrued itraconazole treatment costs for a longer period following the end of the trial. Second, these patients spent more time living with AIDS, hence they spent more on HIV/AIDS-related services and incurred more non-medical expenditures. This cost difference persisted when the cost estimates from the lifetime analysis were combined with those from the within-trial analysis.

*Cost-effectiveness calculations*

Cost and QALY data from the lifetime analysis were then combined with cost and QALY data from the within-trial analysis to calculate two different incremental cost-effectiveness ratios:

1. (Healthcare provider costs and post-trial extrapolation costs for amphotericin minus the same costs for itraconazole) / (Combined QALY data for amphotericin minus the same data for itraconazole)

Undiscounted:

- - ($7,785 - $6,977) / (12.67 – 11.61) = $808 / 1.06
  - Incremental cost-effectiveness ratio (ICER): **$762 per QALY gained**

Health and cost outcomes discounted at 3% per annum:

- - ($7,158 - $6,504) / (8.97 – 8.19) = $654 / 0.77
  - Incremental cost-effectiveness ratio (ICER): **$849 per QALY gained**

1. (Healthcare provider costs and societal costs and post-trial extrapolation costs for amphotericin minus the same costs for itraconazole) / (Combined QALY data for amphotericin minus the same data for itraconazole)

Undiscounted:

- - ($9,562 - $8,909) / (12.67 – 11.61) = $653 / 1.06
  - ICER: **$616 per QALY gained**

Health and cost outcomes discounted at 3% per annum:

- - ($8,882 - $8,378) / (8.97 – 8.19) = $504 / 0.77
  - Incremental cost-effectiveness ratio (ICER): **$655 per QALY gained**

In all cases, when a lifetime analytical perspective is considered, the use of amphotericin as induction therapy increases costs and improves health outcomes, at a cost of $616-762 per QALY gained without discounting, or a cost of $655-$849 per QALY gained when discounting future health and cost outcomes at 3% per annum. Amphotericin therefore remains the cost-effective treatment strategy if these ICERs are compared to the WHO cost-effectiveness threshold of $2,171 per QALY gained, or the threshold of $982 calculated by Woods *et al* for all strategies. Amphotericin is not the cost-effective treatment strategy if these ICERs are compared to the threshold of $144 calculated by Woods *et al*.[11]

Limitations

This analytical extension has some key limitations that should be noted. First, the approach taken to model costs and health outcomes beyond the end of the trial period is simplistic and the results are very sensitive to the assumptions that underlie the analysis. Second, these assumptions are based on a small number of patients in the IVAP study for whom limited follow-up data is available. Third, the only costs that were considered in this analytical extension were those associated with the use of itraconazole until CD4 counts rise above 200 cells/mm^3^, and some treatment and out-of-pocket costs that were associated with the AIDS treatment state. Furthermore, the latter costs were based on data from 2011 which was not adjusted for inflation in each year of the trial.

# Part 4: Patient characteristics

**Table S4.1: Number of patients included in the economic evaluation, by study arm and site**

| **Study site** | **Study arm: N (%)** | | **Total** |
| --- | --- | --- | --- |
|  | **Amphotericin B** | **Itraconazole** |  |
| Hospital for Tropical Diseases | 85 (42%) | 85 (42%) | 170 (42%) |
| National Hospital for Tropical Diseases | 66 (33%) | 63 (31%) | 129 (32%) |
| Bach Mai Hospital | 17 (8%) | 19 (9%) | 36 (9%) |
| Vietnam-Sweden Uong Bi Hospital | 13 (6%) | 12 (6%) | 25 (6%) |
| Viet Tiep General Hospital | 22 (11%) | 23 (11%) | 45 (11%) |
| **Total** | **203** | **202** | **405** |

**Table S4.2: Characteristics of patients included in the economic evaluation (N=405)**

| **Characteristic** | **Study arm: Mean (SD)** | | | **Difference**  **Mean (95%CI)** |
| --- | --- | --- | --- | --- |
|  | **All patients** | **Amphotericin B** | **Itraconazole** |  |
| Age | 34.10 (7.32) | 33.95 (7.16) | 34.26 (7.49) | -0.32 (-1.75 to 1.11) |
| Male sex (proportion) | 0.68 (0.47) | 0.71 (0.46) | 0.65 (0.48) | 0.06 (-0.04 to 0.15) |
| Intravenous drug users (proportion) | 0.31 (0.46) | 0.32 (0.47) | 0.30 (0.46) | 0.02 (-0.07 to 0.11) |

Difference = Amphotericin minus Itraconazole. CI = confidence interval. SD = standard deviation.

**Table S4.3: Demographic characteristics of excluded patients (N=22)**

| **Study site** | **Study arm: N (%)** | | **Total** |
| --- | --- | --- | --- |
|  | **Amphotericin B** | **Itraconazole** |  |
| Hospital for Tropical Diseases | 1 (12.5%) | 0 (0%) | 1 (4.6%) |
| National Hospital for Tropical Diseases | 5 (62.5%) | 9 (64.3%) | 14 (63.6%) |
| Bach Mai Hospital | 1 (12.5%) | 3 (21.4%) | 4 (18.2%) |
| Vietnam-Sweden Uong Bi Hospital | 0 (0%) | 0 (0%) | 0 (0%) |
| Viet Tiep General Hospital | 1 (12.5%) | 2 (14.3%) | 3 (13.6%) |
| **Total** | **8** | **14** | **22** |
| **Characteristic** | **Inclusion status: Mean (SD)** | | **Difference**  **Mean (95%CI)** |
|  | **Included patients** | **Excluded patients** |  |
| Mean year of birth ^ | 1979.26 (7.32) | 1978.36 (8.32) | -0.90 (-4.06 to 2.27) |
| Male sex (proportion) | 0.68 (0.47) | 0.82 (0.39) | 0.14 (-0.06 to 0.34) |
| Intravenous drug users (proportion) | 0.31 (0.46) | 0.41 (0.50) | 0.10 (-0.10 to 0.29) |

CI = confidence interval. SD = standard deviation. ^ not possible to calculate age as all patients have missing admission date.

# Part 5: Resource use by patients during the inpatient and follow-up periods of the trial

**Table S5.1: Healthcare-related resource use per patient at enrolment and during the inpatient period**

| **Study timepoint** | **Resource use item** | **Study arm: Mean (SD)** | | **Difference**  **Mean (95%CI)** |
| --- | --- | --- | --- | --- |
|  |  | **Amphotericin B** | **Itraconazole** |  |
| Enrolment (N=405) | Number of blood culture tests | 1 (0) | 1 (0) | - |
|  | Number of skin smear tests | 0.70 (0.46) | 0.73 (0.44) | -0.02 (-0.11 to 0.06) |
|  | Number of skin culture tests | 0.64 (0.48) | 0.68 (0.47) | -0.04 (-0.14 to 0.05) |
|  | Number of lymph smear tests | 0.06 (0.25) | 0.08 (0.27) | -0.02 (-0.07 to 0.04) |
|  | Number of lymph culture tests | 0.05 (0.02) | 0.05 (0.01) | 0.00 (-0.04 to 0.05) |
|  | Number of other smear tests | 0.03 (0.17) | 0.07 (0.25) | -0.04 (-0.08 to 0.00) |
|  | Number of other culture tests | 0.03 (0.17) | 0.07 (0.26) | -0.04 (-0.09 to -0.00)* |
|  | Number of tuberculosis sputum tests | 1.51 (1.26) | 1.59 (1.28) | -0.08 (-0.32 to 0.17) |
|  | Number of chest x-rays | 0.88 (0.32) | 0.87 (0.34) | 0.02 (-0.05 to 0.08) |
|  | Number of abdominal ultrasounds | 1.26 (0.57) | 1.20 (0.54) | 0.05 (-0.05 to 0.16) |
| Inpatient period, after enrolment (N=405) | Number of blood culture tests | 4.35 (0.96) | 4.06 (1.07) | 0.29 (0.09 to 0.49)** |
|  | Number of chest x-rays | 0.33 (0.64) | 0.23 (0.57) | 0.09 (-0.02 to 0.20) |
|  | Number of Sputum BK tests | 0.07 (0.34) | 0.05 (0.30) | 0.01 (-0.05 to 0.08) |
|  | Number of abdominal ultrasounds | 0.18 (0.51) | 0.15 (0.40) | 0.03 (-0.06 to 0.12) |
|  | Number of other investigations | 1.21 (1.67) | 1.47 (2.05) | -0.26 (-0.62 to 0.11) |
|  | Number of units of blood received | 2.53 (3.29) | 1.81 (2.66) | 0.73 (0.14 to 1.31)* |
|  | Number of platelets received | 0.67 (3.46) | 0.68 (3.00) | -0.01 (-0.65 to 0.62) |

Difference = Amphotericin minus Itraconazole. CI = confidence interval. SD = standard deviation. * = significant at 5% level. ** = significant at 1% level.

**Table S5.2: Healthcare-related resource use per patient during the follow-up period**

| **Resource use item** | **Original dataset** | | | | **Imputed resource use (N=379 ^a^)** | | |
| --- | --- | --- | --- | --- | --- | --- | --- |
|  | **N** | **Study arm: Mean (SD)** | | **Difference**  **Mean (95%CI)** | **Study arm: Mean (SE)** | | **Difference**  **Mean (95%CI)** |
|  |  | **Amphotericin B** | **Itraconazole** |  | **Amphotericin B** | **Itraconazole** |  |
| Number of times patient required emergency care (of any duration) ^b^ | 135 | 0.03 (0.17) | 0.05 (0.21) | -0.02  (-0.08 to 0.05) | - | - | - |
| Proportion of patients who required outpatient care at least once ^b^ | 163 | 0.19 (0.39) | 0.22 (0.42) | -0.03  (-0.16 to 0.09) | - | - | - |
| Number of days of outpatient care required, by patients who required outpatient care at least once ^b^ | 33 | 4.13 (2.20) | 3.72 (2.16) | 0.41  (-1.14 to 1.97) | - | - | - |
| Proportion of patients who required inpatient care at least once ^b^ | 206 | 0.34 (0.48) | 0.47 (0.50) | -0.12  (-0.26 to 0.01) | - | - | - |
| Number of days of inpatient care required, by patients who required inpatient care at least once ^b^ | 85 | 16.61 (10.63) | 14.79 (10.91) | 1.82  (-2.96 to 6.60) | - | - | - |
| Number of skin lesion tests for penicillium marneffei diagnosis | 361 | 0.01 (0.07) | 0.08 (0.30) | -0.08  (-0.12 to -0.03)** | 0.01 (0.01) | 0.08 (0.02) | -0.08  (-0.12 to -0.03)** |
| Number of blood culture tests for penicillium marneffei diagnosis | 361 | 0.02 (0.13) | 0.05 (0.21) | -0.03  (-0.06 to 0.01) | 0.02 (0.01) | 0.05 (0.02) | -0.03  (-0.06 to 0.01) |
| Number of abdominal ultrasounds for penicillium marneffei diagnosis | 361 | 0.01 (0.15) | 0.03 (0.31) | -0.02  (-0.07 to 0.03) | 0.01 (0.01) | 0.03 (0.02) | -0.02  (-0.07 to 0.03) |
| Number of routine blood culture tests | 347 | 1.88 (0.39) | 1.92 (0.38) | -0.04  (-0.12 to 0.04) | 1.85 (0.03) | 1.89 (0.04) | -0.04  (-0.14 to 0.05) |
| Number of routine abdominal ultrasounds | 347 | 0.64 (0.84) | 0.78 (0.87) | -0.14  (-0.32 to 0.04) | 0.68 (0.06) | 0.80 (0.06) | -0.12  (-0.30 to 0.06) |
| Number of routine other tests | 347 | 0.26 (0.60) | 0.38 (0.82) | -0.12  (-0.27 to 0.04) | 0.26 (0.05) | 0.36 (0.06) | 0.10  (-0.25 to 0.05) |

^a^ 26 patients died during the inpatient period. ^b^ These resource use items were costed for all patients (N=405). However, information was not available on how resource use was linked to these costs. Therefore, it was not necessary to impute these resource use items. * = significant at 5% level. ** = significant at 1% level. Difference = Amphotericin minus Itraconazole. CI = confidence interval. SD = standard deviation. SE = standard error.

**Table S5.3: Resource use related to productivity and indirect costs per patient during the inpatient period**

| **Resource use item** | **Original dataset** | | | | **Imputed resource use** | | | |
| --- | --- | --- | --- | --- | --- | --- | --- | --- |
|  | **N** | **Study arm: Mean (SD)** | | **Difference**  **Mean (95%CI)** | **N** | **Study arm: Mean (SE)** | | **Difference**  **Mean (95%CI)** |
|  |  | **Amphotericin B** | **Itraconazole** |  |  | **Amphotericin B** | **Itraconazole** |  |
| Proportion of patients employed | 373 | 0.51 (0.50) | 0.55 (0.50) | -0.04  (-0.14 to 0.06) | 405 | 0.52 (0.04) | 0.54 (0.04) | -0.02  (-0.12 to 0.08) |
| Proportion of patients who lost income due to being hospitalised, for patients who were employed ^a^ | 197 | 0.89 (0.32) | 0.93 (0.26) | -0.05  (-0.13 to 0.04) | - ^a^ | 0.89 (0.03) | 0.93 (0.03) | -0.04  (-0.12 to 0.04) |
| Proportion of patients for whom a carer came to the hospital to take care of the patient | 379 | 0.91 (0.29) | 0.89 (0.32) | 0.02  (-0.04 to 0.08) | 405 | 0.91 (0.02) | 0.88 (0.02) | 0.03  (-0.04 to 0.09) |
| Proportion of patients whose carers lost income while looking after them ^b^ | 334 | 0.56 (0.50) | 0.59 (0.49) | -0.03  (-0.14 to 0.07) | - ^a^ | 0.56 (0.04) | 0.58 (0.04) | 0.03  (-0.13 to 0.08) |
| Proportion of patients who accessed childcare due to their hospitalisation | 371 | 0.35 (0.48) | 0.31 (0.46) | 0.04  (-0.06 to 0.13) | 405 | 0.34 (0.03) | 0.31 (0.03) | 0.03  (-0.06 to 0.13) |
| Number of days of childcare required, for patients who accessed childcare due to their hospitalisation | 119 | 17.77 (6.44) | 18.70 (12.75) | -0.93  (-4.55 to 2.70) | - ^a^ | 17.69 (0.79) | 18.46 (1.59) | -0.77  (-4.17 to 2.64) |

^a^ Sample size varies across imputations. ^b^ Out of those who required a carer. Difference = Amphotericin minus Itraconazole. CI = confidence interval. SD = standard deviation. SE = standard error.

**Table S5.4: Resource use related to** **productivity and indirect costs per patient during the follow-up period**

| **Resource use item** | **Original dataset** | | | | **Imputed resource use** | | | |
| --- | --- | --- | --- | --- | --- | --- | --- | --- |
|  | **N** | **Study arm: Mean (SD)** | | **Difference**  **Mean (95%CI)** | **N** | **Study arm: Mean (SE)** | | **Difference**  **Mean (95%CI)** |
|  |  | **Amphotericin B** | **Itraconazole** |  |  | **Amphotericin B** | **Itraconazole** |  |
| Proportion of patients employed | 305 | 0.53 (0.50) | 0.51 (0.50) | 0.02  (-0.09 to 0.13) | 379 ^a^ | 0.50 (0.04) | 0.47 (0.05) | 0.03  (-0.08 to 0.15) |
| Number of days taken off sick, for patients who were employed | 156 | 12.10 (17.77) | 15.66 (30.28) | -3.56  (-11.33 to 4.20) | - ^b^ | 11.81 (1.90) | 16.29 (4.16) | -4.48  (-13.30 to 4.33) |
| Proportion of patients for whom a carer came to the hospital to take care of the patient | 305 | 0.39 (0.49) | 0.49 (0.50) | -0.10  (-0.21 to 0.01) | 379 ^a^ | 0.40 (0.04) | 0.55 (0.04) | -0.15  (-0.26 to -0.04)* |
| Proportion of patients whose carers lost income while looking after them ^c^ | 131 | 0.52 (0.50) | 0.56 (0.50) | -0.05  (-0.22 to 0.13) | - ^b^ | 0.52 (0.07) | 0.56 (0.08) | -0.04  (-0.25 to 0.16) |
| Proportion of patients who accessed childcare due to their hospitalisation | 293 | 0.24 (0.43) | 0.29 (0.44) | -0.05  (-0.16 to 0.05) | 379 ^a^ | 0.24 (0.04) | 0.27 (0.04) | -0.03  (-0.14 to 0.08) |
| Number of days of childcare required, for patients who accessed childcare due to their hospitalisation | 78 | 14.42 (14.42) | 20.38 (26.99) | -5.96  (-15.96 to 4.04) | - ^b^ | 14.55 (2.46) | 21.51 (4.79) | -6.96  (-17.96 to 4.04) |

^a^ 26 patients died during the inpatient period. ^b^ Sample size varies across imputations. ^c^ Out of those who required a carer. * = significant at 5% level. Difference = Amphotericin minus Itraconazole. CI = confidence interval. SD = standard deviation. SE = standard error.

Commentary

There were few significant differences in resource use by study arm across the inpatient and follow-up periods of the trial, both for healthcare-related resource use and also for resource use related to productivity and indirect costs.

In terms of healthcare-related resource use, there was a significant difference in the mean number of blood culture tests per patient during the inpatient period, with patients in the amphotericin arm having 0.29 additional tests (95% confidence interval 0.09 to 0.49). Patients in the amphotericin arm also received more units of blood (2.53 units versus 1.81 units, mean difference 0.73 (95% confidence interval 0.14 to 1.31)). During the follow-up period, healthcare-related resource use was generally higher for patients in the itraconazole arm (particularly the proportion of patients who required inpatient or outpatient care at least once), however these differences were not statistically significant. The only difference was in the mean number of skin lesion tests for penicillium marneffei diagnosis. This was 0.01 for patients in the amphotericin arm and 0.08 for patients in the itraconazole arm (mean difference -0.08; 95% confidence interval -0.12 to -0.03).

In terms of resource use related to productivity and indirect costs, the only difference was in the mean proportion of patients for whom a carer came to the hospital to take care of the patient. This was 0.40 in the amphotericin arm and 0.55 in the itraconazole arm (mean difference -0.15; 95% confidence interval -

0.26 to -0.04).

# Part 6: Costs in Vietnamese Dong

**Table S6.1: Per patient costs from a healthcare provider perspective, in 2016 Vietnamese Dong**

| **Study timepoint** | **Cost category** | **Study arm: Mean (SE) (đ)** | | **Difference**  **Mean (95%CI) (đ)** |
| --- | --- | --- | --- | --- |
|  |  | **Amphotericin B** | **Itraconazole** |  |
| Inpatient period  (N=405) | Diagnosis | 3,162,749 (117,856) | 3,130,819 (114,093) | 31,929 (-290,573 to 354,432) |
|  | Inpatient stays | 1,338,295 (103,339) | 1,247,475 (62,065) | 90,820 (-146,436 to 328,077) |
|  | Drugs | 6,166,003 (606,053) | 6,382,801 (643,527) | -216,798 (-1,954,371 to 1,520,775) |
|  | Procedures and operations | 87,176 (26,117) | 71,343 (14,147) | 15,833 (-42,639 to 74,304) |
|  | Blood transfusions | 1,661,483 (169,616) | 1,291,816 (166,345) | 369,667 (-97,400 to 836,734) |
|  | Platelets | 389,438 (137,114) | 580,741 (203,770) | -191,302 (-673,692 to 291,087) |
|  | Other costs | 766,188 (49,468) | 655,030 (53,431) | 111,158 (-31,962 to 254,278) |
|  | Trial drugs | 5,392,957 (120,549) | 1,317,238 (66,909) | 4,075,719 (3,804,320 to 4,347,119)** |
|  | **Total – inpatient period** | **18,964,576 (918,317)** | **14,677,183 (907,280)** | **4,287,393 (1,749,495 to 6,825,291)**** |
| Follow-up period  (N=379) | Trial drugs | 7,666,837 (161,880) | 7,362,634 (177,490) | 304,204 (-167,949 to 776,357) |
|  | Hospital costs | 2,640,155 (598,337) | 6,549,665 (1,426,917) | -3,909,510 (-6,962,144 to -856,876)* |
|  | Other test cost | 3,825 (2,085) | 19,295 (5,003) | -15,470 (-26,099 to -4,842)** |
|  | Other diagnostic costs | 365,509 (15,151) | 429,677 (24,392) | -64,167 (-120,641 to -7,693)* |
|  | **Total – follow-up period** | **10,676,326 (623,721)** | **14,361,270 (1,426,761)** | **-3,684,944 (-6,757,081 to -612,807)*** |
|  | **Total – inpatient and follow-up period** | **29,640,902 (1,198,383)** | **29,038,452 (1,809,915)** | **602,450 (-3,664,831 to 4,869,730)** |

* = significant at 5% level. ** = significant at 1% level. Difference = Amphotericin minus Itraconazole. CI = confidence interval. SE = standard error.

**Table S6.2: Mean non-health care costs per patient, in 2016 Vietnamese Dong**

| **Study timepoint** | **Cost category** | **Study arm: Mean (SE) (đ)** | | **Difference**  **Mean (95%CI) (đ)** |
| --- | --- | --- | --- | --- |
|  |  | **Amphotericin B** | **Itraconazole** |  |
| Inpatient period  (N=405) | Patient out-of-pocket costs | 3,291,120 (260,741) | 3,491,460 (303,788) | -200,340 (-987,079 to 586,399) |
|  | Patient travel costs | 710,942 (83,938) | 836,811 (111,681) | -125,868 (-399,423 to 147,686) |
|  | Patient lost income | 1,995,749 (268,054) | 2,185,222 (365,742) | -189,473 (-1,082,357 to 703,411) |
|  | Carer lost income | 1,435,921 (162,100) | 1,480,619 (144,100) | -44,698 (-472,124 to 382,728) |
|  | Childcare costs | 578,595 (63,211) | 539,547 (73,446) | 39,048 (-151,420 to 229,517) |
|  | **Total – inpatient period** | **8,012,328 (504,239)** | **8,533,659 (633,563)** | **-521,332 (-2,112,816 to 1,070,153)** |
| Follow-up period  (N=379) | Patient out-of-pocket costs | 1,671,376 (366,307) | 1,533,012 (272,464) | 138,364 (-763,802 to 1,040,530) |
|  | Patient travel costs | 1,408,306 (175,905) | 1,364,384 (151,667) | 43,922 (-415,746 to 503,590) |
|  | Patient lost income | 983,116 (174,948) | 1,448,979 (410,318) | -465,862 (-1,336,818 to 405,093) |
|  | Carer lost income | 596,286 (175,986) | 742,735 (182,702) | -146,448 (-650,711 to 357,815) |
|  | Childcare costs | 328,594 (72,793) | 510,981 (144,036) | -182,386 (-499,401 to 134,629) |
|  | **Total – follow-up period** | **4,987,679 (536,520)** | **5,600,090 (689,376)** | **-612,411 (-2,309,207 to 1,084,386)** |
|  | **Total – inpatient and follow-up period** | **13,000,007 (782,070)** | **14,133,749 (1,018,559)** | **-1,133,742 (-3,640,989 to 1,373,504)** |

* = significant at 5% level. Difference = Amphotericin minus Itraconazole. CI = confidence interval. SE = standard error.

**Table S6.3: Total societal (healthcare and non-healthcare) costs per patient, in 2016 Vietnamese Dong**

| **Cost category** | **Study arm: Mean (SE) (đ)** | | **Difference**  **Mean (95%CI) (đ)** |
| --- | --- | --- | --- |
|  | **Amphotericin B** | **Itraconazole** |  |
| Inpatient period | 26,976,903 (1,144,503) | 23,210,842 (1,301,848) | 3,766,061 (359,200 to 7,172,923)* |
| Follow-up period | 15,664,005 (946,358) | 19,961,360 (1,684,433) | -4,297,354 (-8,073,043 to -521,666)* |
| **Total – inpatient and follow-up period** | **42,640,909 (1,642,959)** | **43,172,202 (2,308,615)** | **-531,293 (-6,080,974 to 5,018,388)** |

* = significant at 5% level. Difference = Amphotericin minus Itraconazole. CI = confidence interval. SE = standard error.

# Part 7: Quality of life and health outcomes results

**Table S7.1: EQ-5D-5L domain frequencies, by study arm and timepoint**

| **Study timepoint** | **EQ-5D-5L dimension** | **Percentage of each study arm reporting EQ-5D-5L levels 1 to 5 by dimension ^a^** | | | | | | | | | |
| --- | --- | --- | --- | --- | --- | --- | --- | --- | --- | --- | --- |
|  |  | **Amphotericin B** | | | | | **Itraconazole** | | | | |
|  |  | **1** | **2** | **3** | **4** | **5** | **1** | **2** | **3** | **4** | **5** |
| Baseline | Mobility | 26.2% | 33.9% | 13.3% | 19.0% | 7.7% | 27.5% | 28.5% | 19.2% | 13.5% | 11.4% |
|  | Self-care | 38.0% | 28.2% | 12.8% | 11.8% | 9.2% | 32.6% | 27.5% | 11.4% | 16.1% | 12.4% |
|  | Usual activity | 29.2% | 31.3% | 18.5% | 13.9% | 7.2% | 30.6% | 26.4% | 13.0% | 18.1% | 12.0% |
|  | Pain/discomfort | 18.0% | 35.9% | 24.1% | 19.0% | 3.1% | 16.6% | 35.2% | 24.4% | 20.7% | 3.1% |
|  | Anxiety/depression | 32.8% | 34.4% | 22.6% | 9.2% | 1.0% | 26.4% | 32.6% | 24.9% | 15.0% | 1.0% |
| Discharge | Mobility | 62.9% | 25.8% | 7.5% | 1.6% | 2.2% | 58.8% | 30.5% | 3.4% | 5.7% | 1.7% |
|  | Self-care | 71.5% | 20.4% | 4.3% | 1.6% | 2.2% | 67.2% | 19.8% | 5.1% | 5.7% | 2.3% |
|  | Usual activity | 62.9% | 29.0% | 5.4% | 2.2% | 0.5% | 59.3% | 29.4% | 6.8% | 2.8% | 1.7% |
|  | Pain/discomfort | 58.6% | 33.3% | 5.9% | 2.2% | 0.0% | 58.8% | 31.1% | 7.3% | 2.3% | 0.6% |
|  | Anxiety/depression | 64.0% | 33.3% | 1.6% | 1.1% | 0.0% | 59.9% | 30.5% | 6.2% | 2.3% | 1.1% |
| End of follow-up period | Mobility | 90.8% | 8.0% | 0.6% | 0.6% | 0.0% | 92.2% | 7.1% | 0.7% | 0.0% | 0.0% |
|  | Self-care | 95.1% | 3.7% | 1.2% | 0.0% | 0.0% | 96.1% | 3.9% | 0.0% | 0.0% | 0.0% |
|  | Usual activity | 93.9% | 5.5% | 0.6% | 0.0% | 0.0% | 92.9% | 5.8% | 0.7% | 0.7% | 0.0% |
|  | Pain/discomfort | 86.5% | 9.8% | 3.1% | 0.6% | 0.0% | 79.4% | 18.7% | 1.3% | 0.7% | 0.0% |
|  | Anxiety/depression | 87.1% | 10.4% | 2.5% | 0.0% | 0.0% | 78.1% | 17.4% | 4.5% | 0.0% | 0.0% |

^a^ 1 = No problems, 2 = Slight problems, 3 = Moderate problems, 4 = Severe problems, 5 = Extreme problems.

**Table S7.2: Quality-adjusted life years (QALYs) per patient, by study arm**

| **Analysis** | **Study arm: Mean (SE)** | | **Naïve Difference:**  **Mean (95%CI)** | **Difference adjusted for baseline quality of life: Mean (95% CI)** |
| --- | --- | --- | --- | --- |
|  | **Amphotericin B** | **Itraconazole** |  |  |
| *Quality of life at baseline* | *0.570 (0.024)* | *0.519 (0.026)* | *0.051 (-0.019 to 0.121)* | - |
| Within-trial analysis | 0.384 (0.010) | 0.350 (0.011) | 0.034 (0.004 to 0.063)* | *0.027 (0.002 to 0.052)** |
| Alternative approach 1 | 0.403 (0.010) | 0.367 (0.012) | 0.036 (0.005 to 0.066)* | *0.030 (0.003 to 0.056)** |
| Alternative approach 2 | 0.378 (0.009) | 0.344 (0.011) | 0.034 (0.005 to 0.063)* | *0.028 (0.003 to 0.053)** |

* = significant at 5% level.

Note: In the **within-trial analysis**, we assumed that the first EQ-5D-5L survey was completed on the admission date, the second survey was completed on the discharge date, and the third survey was completed at the end of the follow-up period. In **alternative approach 1**, we assumed that the three EQ-5D-5L surveys were completed at the same timepoints by each patient (Day 0, then 14 days after admission, then 182 days after admission). In **alternative approach 2**, we calculated quality of life using the recorded dates for each survey.

Commentary

**Table S7.1** reports the proportion of each study arm reporting EQ-5D-5L levels 1 to 5 by dimension (with a level of 1 indicating no problems and a level of 5 indicating extreme problems). At baseline there were no differences between the two arms in terms of pain/discomfort. More amphotericin patients (26.7%) reported severe or extreme mobility problems compared to itraconazole patients (24.9%), whereas more itraconazole patients reported severe or extreme problems in the self-care, usual activity and anxiety/depression dimensions (28.5% vs. 21.0%, 30.1% vs. 21.1%, and 16.0% vs. 10.2%, respectively). At discharge, more amphotericin patients reported either no or slight problems for all dimensions except for mobility (89.3% vs. 88.7% in favour of itraconazole). At the end of the follow-up period, the percentage of patients reporting no problems in the mobility, self-care and usual activity dimensions was approximately similar. However, more amphotericin patients reported no problems for the pain/discomfort and anxiety/depression dimensions (86.5% vs. 79.4% and 87.1% vs. 78.1%, respectively).

**Table S7.2** presents the health outcome results (in terms of QALYs) by study arm. Patients in the amphotericin arm gained more QALYs than patients who received itraconazole (baseline-adjusted mean difference 0.027; 95% confidence interval 0.002 to 0.052). This difference is consistent across assumptions made across sensitivity analyses regarding the calculation of QALYs. Given this, the within-trial analysis QALY estimates were used in the main analysis.

We also measured health outcomes using life-years as the outcome measure, as part of the within-trial analysis. Patients in the amphotericin arm accrued 0.468 life-years across the trial period (95% confidence interval 0.459 to 0.476) and patients in the itraconazole arm accrued 0.451 life-years (95% confidence interval 0.439 to 0.464). The mean difference was 0.016 life-years which was statistically significant at a 5% level (95% confidence interval 0.001 to 0.031). The cost per life-year gained for amphotericin versus itraconazole was $5,079.

# Part 8: Cost-effectiveness acceptability curves

**Figure S8.1: Cost-effectiveness acceptability curves indicating the probability of each treatment being the cost-effective treatment strategy at a range of values for the cost-effectiveness threshold**

**Figure S8.2: Cost-effectiveness acceptability frontier indicating the threshold at which Amphotericin is the cost-effective treatment strategy**

# Part 9: Sensitivity analysis results

**Table S9.1: Variations in the unit costs of the trial drugs, in 2016 United States Dollars**

**Adjusted for difference in baseline quality of life between arms**

| **Variation in drug unit cost** | **Healthcare provider perspective** | | | | | | **Societal perspective** | | | | | |
| --- | --- | --- | --- | --- | --- | --- | --- | --- | --- | --- | --- | --- |
|  | **Incremental costs ($)** | **Incremental QALYs** | **Cost per QALY gained ($)** | **Probability of amphotericin being cost-effective at different values of the cost-effectiveness threshold** | | | **Incremental costs ($)** | **Incremental QALYs** | **Cost per QALY gained ($)** | **Probability of amphotericin being cost-effective at different values of the cost-effectiveness threshold** | | |
|  |  |  |  | **Lower Woods threshold ($144)** | **Upper Woods threshold ($982)** | **WHO threshold ($2,171)** |  |  |  | **Lower Woods threshold ($144)** | **Upper Woods threshold ($982)** | **WHO threshold ($2,171)** |
| Assumed cost ^a^ | 82 | 0.027 | 3,013 | 39% | 42% | 46% | -72 | 0.027 | N/A ^b^ | 58% | 60% | 63% |
| **Amphotericin** | | | | | | | | | | | | |
| Low Cost | -266 | 0.027 | N/A ^b^ | 82% | 84% | 87% | -420 | 0.027 | N/A ^b^ | 87% | 88% | 90% |
| High Cost | 430 | 0.027 | 15,808 | 8% | 9% | 11% | 276 | 0.027 | 10,148 | 23% | 25% | 28% |
| **Itraconazole** | | | | | | | | | | | | |
| Low Cost | 131 | 0.027 | 4,829 | 33% | 35% | 40% | -23 | 0.027 | N/A ^b^ | 53% | 55% | 58% |
| High Cost | 33 | 0.027 | 1,198 | 45% | 48% | 53% | -121 | 0.027 | N/A ^b^ | 63% | 65% | 68% |

**Not adjusted for difference in baseline quality of life between arms**

| **Variation in drug unit cost** | **Healthcare provider perspective** | | | | | | **Societal perspective** | | | | | |
| --- | --- | --- | --- | --- | --- | --- | --- | --- | --- | --- | --- | --- |
|  | **Incremental costs ($)** | **Incremental QALYs** | **Cost per QALY gained ($)** | **Probability of amphotericin being cost-effective at different values of the cost-effectiveness threshold** | | | **Incremental costs ($)** | **Incremental QALYs** | **Cost per QALY gained ($)** | **Probability of amphotericin being cost-effective at different values of the cost-effectiveness threshold** | | |
|  |  |  |  | **Lower Woods threshold ($144)** | **Upper Woods threshold ($982)** | **WHO threshold ($2,171)** |  |  |  | **Lower Woods threshold ($144)** | **Upper Woods threshold ($982)** | **WHO threshold ($2,171)** |
| Assumed cost ^a^ | 82 | 0.034 | 2,438 | 39% | 43% | 48% | -72 | 0.034 | N/A ^b^ | 58% | 61% | 65% |
| **Amphotericin** | | | | | | | | | | | | |
| Low Cost | -266 | 0.034 | N/A ^b^ | 83% | 85% | 88% | -420 | 0.034 | N/A ^b^ | 87% | 88% | 90% |
| High Cost | 430 | 0.034 | 12,791 | 8% | 9% | 12% | 276 | 0.034 | 8,211 | 23% | 26% | 29% |
| **Itraconazole** | | | | | | | | | | | | |
| Low Cost | 131 | 0.034 | 3,907 | 33% | 36% | 42% | -23 | 0.034 | N/A ^b^ | 53% | 56% | 60% |
| High Cost | 33 | 0.034 | 969 | 46% | 49% | 55% | -121 | 0.034 | N/A ^b^ | 63% | 66% | 69% |

Incremental figures = amphotericin minus itraconazole. ^a^ Low cost for amphotericin = $10 per 50mg vial; high cost for amphotericin = $30 per 50mg vial; low cost for itraconazole = $0.44 per 100mg tablet; high cost for itraconazole = $1.33 per 100mg tablet. ^b^ Amphotericin reduces costs and improves health outcomes.

Commentary

**Table S9.1** reports the results of the sensitivity analyses around drug costs. Variations in the cost of amphotericin lead to notable changes in the cost-effectiveness results. When a cost that is 50% of the assumed value is applied to the baseline-adjusted analysis, amphotericin becomes cost-saving from both a healthcare provider and societal perspective. The probability that amphotericin is the cost-effective treatment strategy from a healthcare provider perspective increases from 46% to 87% at the WHO cost-effectiveness threshold of $2,171. From a societal perspective, the probability that amphotericin is the cost-effective treatment strategy increases from 63% to 90% at the same threshold. When a cost that is 150% of the assumed value is applied to the baseline-adjusted analysis, amphotericin is no longer the cost-effective treatment strategy. From a healthcare provider perspective, the incremental cost-effectiveness ratio in this scenario is $15,808 per QALY gained (95%CI: cost saving to $98,495 per QALY gained). From a societal perspective, the incremental cost-effectiveness ratio in this scenario is $10,148 per QALY gained (95%CI: cost saving to $87,796 per QALY gained).

Changes in the cost of itraconazole have a smaller impact on the cost-effectiveness of amphotericin. When a cost that is 50% of the assumed value is applied to the baseline-adjusted analysis, amphotericin has a 40% chance of being the cost-effective treatment strategy from a healthcare provider perspective at the WHO threshold of $2,171. In this scenario, the incremental cost-effectiveness ratio is estimated to be $4,829 per QALY gained (95%CI: cost saving to $59,496 per QALY gained). From a societal perspective amphotericin remains cost-saving, but the probability that amphotericin is the cost-effective treatment strategy reduces from 63% to 58% at the same threshold. When a cost that is 150% of the assumed value is applied, the cost-effectiveness of amphotericin improves, with the incremental cost-effectiveness ratio reducing to $1,198 per QALY gained (95%CI: cost saving to $45,549 per QALY gained) from a healthcare provider perspective, and with amphotericin remaining cost-saving from a societal perspective.

# References

1. General Statistics Office of Vietnam. Available at: <https://www.gso.gov.vn/default_en.aspx?tabid=783>.

2. American Chamber of Commerce Vietnam. Minimum Wage Adjustment for 2012 to be effective from Oct 5, 2011. Available at: <https://www.amchamvietnam.com/minimum-wage-adjustment-for-2012-to-be-effective-from-oct-5-2011/>. Accessed 02/05/2017.

3. Vietnam Briefing. Minimum Wages in Vietnam to Increase from January 2013. Available at: <https://www.vietnam-briefing.com/news/regionbased-minimum-wages-increase-january-2013.html/>. Accessed 02/05/2017.

4. WageIndicator. Minimum Wage – Vietnam. Available at: <https://wageindicator.org/salary/minimum-wage/vietnam>. Accessed 02/05/2017.

5. Faria R, Gomes M, Epstein D, White IR. A Guide to Handling Missing Data in Cost-Effectiveness Analysis Conducted Within Randomised Controlled Trials. PharmacoEconomics **2014**; 32(12): 1157-70.

6. World Health Organisation: Global Health Observatory data repository. Available at: <http://apps.who.int/gho/data/?theme=main&vid=61830>.

7. Mills EJ, Bakanda C, Birungi J, et al. Life expectancy of persons receiving combination antiretroviral therapy in low-income countries: A cohort analysis from uganda. Annals of Internal Medicine **2011**; 155(4): 209-16.

8. Tran BX, Ohinmaa A, Nguyen LT. Quality of life profile and psychometric properties of the EQ-5D-5L in HIV/AIDS patients. Health and Quality of Life Outcomes **2012**; 10(1): 132.

9. Vietnam Ministry of Health. Guidelines for HIV/AIDS Diagnosis and Treatment. Published with Decision No. 3003/Qð-BYT dated 19/8/2009 of the Minister of Health. Ha Noi, **2009**.

10. Tran BX, Duong AT, Nguyen LT, et al. Financial burden of health care for HIV/AIDS patients in Vietnam. Tropical Medicine & International Health **2013**; 18(2): 212-8.

11. Woods B, Revill P, Sculpher M, Claxton K. Country-Level Cost-Effectiveness Thresholds: Initial Estimates and the Need for Further Research. Value in Health **2016**; 19(8): 929-35.
